# Liquid–Gas Phase Transition Actuator: Rejuvenation Procedure Extended and Open-Air Performance

**DOI:** 10.3390/polym17010020

**Published:** 2024-12-25

**Authors:** Igor Bezsudnov, Alina Khmelnitskaia, Aleksandra Kalinina, Kristina Monakhova, Sergey Ponomarenko

**Affiliations:** Enikolopov Institute of Synthetic Polymeric Materials of Russian Academy of Sciences (ISPM RAS), Profsoyuznaya Str. 70, 117393 Moscow, Russia; alina.khmelnitskaya@ispm.ru (A.K.); kalinina@ispm.ru (A.K.); k.myagkova@ispm.ru (K.M.); ponomarenko@ispm.ru (S.P.)

**Keywords:** soft actuator, silicone foam, alcohols, rejuvenation procedure, open-air performance, multi-cyclic actuation, performance prediction

## Abstract

To achieve the actuation of silicone-based foamed composites, a liquid–gas phase transition of the liquid captured in its pores is employed. The uncertainty of key parameters for a single or sequential open-air performance of such soft actuators limits their application. To define the main characteristics of the composites, in this work, two functions of the liquid there were separated: the pore-forming agent (FPA) and working liquid (WL). It was demonstrated that the composites can be fabricated using either ethanol or methanol as the PFA, while any of the C1-C4 alcohols can be used as the WL. The results of the sequential actuation tests of the composites revealed that pore formation depends on the composite viscosity during curation, while their expansion in single heat experiments can be approximated by a unified linear relation. Based on a Mendeleev–Clapeyron equation, the qualitative model for predicting the actuator strain is proposed. It was found that the composites with C3–C4 alcohols as the WL outperform ethanol-containing composites on the number of cycles survived under open-air conditions. These findings pave the way to control the operation of soft actuators by manipulating WL variation and PFA content during the composite cure to set the operation temperature and degree of expansion of pre-formed actuators.

## 1. Introduction

A key challenge in soft robotics now is the development of autonomous soft actuators (or artificial muscles) with electric operation and a high density of stored energy. An extensive list of such modern technologies is presented in many reviews devoted to various types of soft robotics actuators and its applications [1,2,3], current progress, and future perspectives [4,5,6].

Historically, actuators were driven mechanically, pneumatically [7], or hydraulically [8]. The first widely known flexible actuator was the McKibben pneumatic muscle [9], created in the early 1950s of the last century, and this type of actuator is still in use. However, the so-called electroactive flexible polymer actuators are the most prospective, since they use an electric field to be actuated, while the mechanical properties of such materials are similar to animal or human muscles. Actuators operating in a liquid medium can be fabricated based on ionic polymer–metal composites, which are thoroughly described in [10] and in recent reviews [11,12]. Ionic polymer gels are presented in detail in [13] and their current advances as an actuator material are explored in [14,15]. Conducting polymers was proposed initially in [16] and current developments are considered in [17,18]. Field-activated polymers, such as polymer electrets [19], electrostrictive graft copolymers [20,21], ferroelectric polymers [22,23], and relaxor polymers [22] as well as liquid crystal elastomers [24] that can be used as actuators [25,26], can function in the air environment. The working principle of dielectric elastomer actuators was first demonstrated by W. Röntgen [27] on a soft and flexible rubber membrane, with electrical contacts deposited on its surface. They are actuated when an electric field is applied, and the membrane is compressed in thickness and stretched in-plane. Pioneering publications describing the construction of this type of actuator are [28,29,30], and contemporary advances can be found in [31,32]. New types of polymers, composites, or devices are constantly emerging; for example, some use animal muscle cells or plant cells [33,34]. All the aforementioned kinds of actuators have some drawbacks that limit their autonomy operations; the dielectric elastomer actuator needs an additional mandatory high-voltage unit (>1 kV), the conductive electroactive polymers require a leaky electrolyte environment, the shape memory alloys deliver only low deformations (<10%) that limit their application, and the hydraulic or pneumatic actuators require external compressors and pressure control.

The actuators based on various flexible functional materials look very promising. One example of such a material is a silicone composite containing inclusions of another material that undergoes solid–liquid or liquid–gas phase transition with the temperature change. Such an actuator is thermally or thermoelectrically actuated, while the latter is more convenient. A number of methods can be used to create a pore structure in the polydimethylsiloxane (PDMS) matrix during its cure. In reviews [35,36,37], one can find a comprehensive list of such methods (even used in sensing technology). The addition of ammonium carbonate or bicarbonate [38,39,40] is used to form a porous structure in cases where the curing process proceeds at 50–70 °C when it decomposes producing gases. The sugar or salt added to a curing PDMS matrix [41,42,43] do not change their own shape or state, but after curing the composite, they can be dissolved in water and, therefore, removed from the formed pores. Also, the water itself forms pores in the silicone matrix [44,45]. However, all the abovementioned techniques and the others mentioned in [35,36,37] have a similar intrinsic feature; the foam is a kind of percolation structure inside the PDMS matrix instead of isolated pores.

An example of a composite using a solid–liquid phase transition with a PDMS matrix with isolated paraffin inclusions distributed over it is presented in [46]. The composite has a rather high stored energy, but quite small deformations (about 3%). At present, some materials of this kind are already industrially used as passive temperature actuators and act as temperature fuses, for instance, in motor cooling systems, hydraulic lines, etc. [47]

The perspective composites can use a liquid–gas transition in such a separated distributed inclusion, showing rather large deformations with the sufficiently high density of the stored energy. A soft PDMS composite matrix with closed pores (microbubbles) containing ethanol was proposed by A. Miryev et al. as “a single easily prepared soft robust material that combines the elastic properties of a polymeric matrix and the extreme volume change of a fluid upon liquid–vapor transition” [48]. The matrix was the commercially available PDMS Ecoflex 00-50 (Smooth-On, Macungie, PA, USA).

The composite preparation included the addition of ethanol to the PDMS composition. During the curing process, the added ethanol released vapor inside the volume of the polymerizing matrix, forming spherical cavities (pores) where the ethanol was eventually captured. Therefore, it can be said that a two-component composite is produced in the form of a PDMS matrix, with the cavities or pores filled with ethanol in the equilibrium of the liquid and gas phases [48]. “The material combines a high strain (up to 900%) and correspondingly high stress (up to 1.3 MPa) with low density (0.84 g cm^−3^). Along with its extremely low cost (about 3 cents per gram), simplicity of fabrication, and environment-friendliness, these properties could enable new kinds of electrically driven entirely soft robots” [48]. In other words, they produce the PDMS composite using a certain amount of ethanol as a pore-forming agent (PFA), and also use ethanol as a working substance or working liquid (WL) that undergoes the liquid–gas transition.

The material was subjected to a fairly detailed investigation in [49], which included the following: a microstructure study, ethanol evaporation analysis by the DSC–TGA–MS complex, mechanical tests of the composite in the block-force mode in a sealed cylindrical test cell, and more.

It should be emphasized that it is the pores that make this composite possible to expand when heated, because initially they hold a working liquid in the equilibrium liquid–gas state in the composite. Also, it is the pores where liquid–gas transition occurs, and finally, the volume where the gas is expanding, pushing the actuator body to extend.

One can state that the advantages of the actuators based on composites employing a liquid–gas phase transition are the ease of manufacture, the simplicity of actuation (thermal heating, including electrical heating), inherent flexibility, and the ability to have an arbitrary shape, as well as the possibility of using additive technologies [50,51] for the manufacturing of the ready-to-use actuator. Regarding the actuators proposed in [48,50,51], for instance, grips are designed for open-air operation, although there is a lack of information on the long-term actuator performance in the open air. At the same time, such results are well known for a sealed environment operation like the block-force mode. This problem was partially solved [52] when a procedure for the composite performance recovery was proposed, called rejuvenation. It is based on the fact that the diffusion of ethanol (as the WL) through the PDMS matrix is quite high. The composite sample is put in ethanol overnight, during which time the pores gain inside an equilibrium mixture of ethanol in the gas and liquid phases.

The source of another discovered shortcoming for this type of actuator is based on the relatively low thermal conductivity of silicones, which calls for the modification and optimization of the composite heating technologies. The originally proposed scheme of heating [48] implies a high-resistance wire paved inside the volume of the composite (this way will be called later “internal heating”). Due to the high density of heat release on the surface of a thin wire, silicone is degrading. The modifications of the internal heating using a thick central rod [53] or conductive fabric heating [54] were proposed. The actuation is also possible by heating the outer surface of a sample (“external heating”), which will be used further in the work. Induction heating was also investigated [55]. Some increase in the heating rate was also achieved by adding heat-conducting inclusions—nanodiamonds [56]—in a concentration that did not significantly affect the mechanical properties of the resulting composite. The same approach to the actuation acceleration was used by Z. Liu et al. [57], who used a mixture of graphene oxide/gold nanoparticles. It seems that the other highly thermally conductive and sufficiently chemically inert fillers can play a similar role.

This work describes a comprehensive investigation of foamed silicone composite actuators employing liquid–gas phase transition. The uncertainty of key parameters for the single or sequential open-air performance of such composites limits the usage of constructions made of such a type of actuators. For the first time, the formation of foamed silicone composites using methanol as a pore-forming agent is demonstrated. The so-called rejuvenation procedure is extended by applying various alcohols as a working liquid, which undergo liquid–gas phase transition. It is found that the specific content of the working liquids in the composite as well as the deformation gained by the actuator linearly depend on the concentration of the pore-forming agent used during the composite preparation.

## 2. Materials and Methods

### 2.1. Materials

The platinum-catalyzed two-part silicone Ecoflex 00-50 (Smooth-On, Macungie, PA, USA) was used as a composite matrix, and the following alcohols were employed both as a pore-forming agent and as a composite working liquid to undergo liquid–gas phase change: ethanol (96%), methanol (99.5%), 1-propanol (99.5%), isopropanol, and butanol were reagent grade (EKOS-1, Moscow, Russia).

### 2.2. Foamed Silicone Preparation

**Figure 1:**
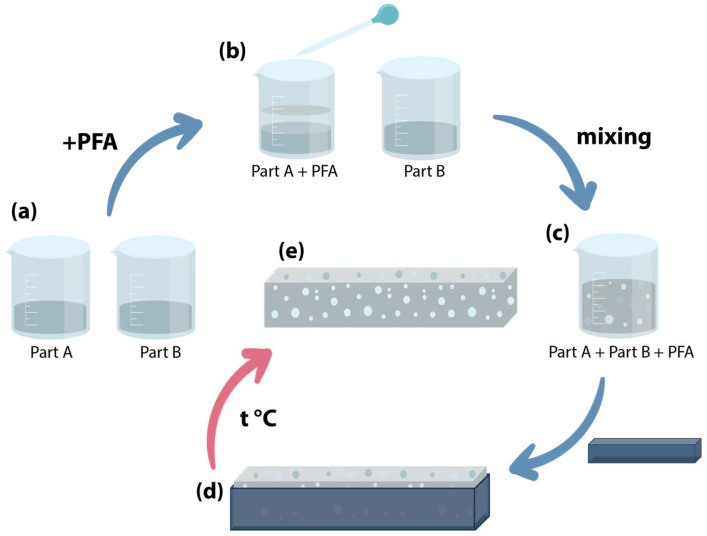
Foamed silicone preparation procedure. (**a**) Ecoflex 00-50 components A and B, (**b**) the PFA addition to component A, (**c**) mixing components, (**d**) mold with composite when curing, (**e**) ready composite treated in heating cabinet.

The sequence of the foamed composite fabrication is shown schematically in Figure 1. The images of the composite preparation are given in Supplementary Materials Figure S1. The Ecoflex 00-50 components A and B are to be mixed at the 1:1 ratio (Figure 1a). To fabricate the foamed composite material, the PFA is added to the silicone component A (Figure 1b) and manually stirred for 1 min. The PFA amount is 3, 5, 10, 15, 20, 25, 30, and 35 vol.% relative to the full A+B composite volume, and then manually mixed with the component B for 1 min (Figure 1c).

The mixture was cast into molds (Figure 1d) made by a 3D printer using ABS (acrylonitrile butadiene styrene) or PLA (polylactic acid) polymer. Two types of molds were used: 20 mm dia. mold (for WL content investigation) and 10 × 10 mm section mold with the 40 mm length for use in the PARUS device (see below) for expansion testing.

The cast material takes up at least three hours to cure at room temperature. Once the silicone had cured, the ready specimen was removed from the mold and treated overnight in the heater cabinet (Figure 1e) at a temperature of 15 °C less than the boiling point of the PFA used to initially remove the preparation solvent from the pores of the composite. The samples that have been used in all the following experiments underwent a rejuvenation procedure [52] with the working liquid. The necessity of the initial rejuvenation is to have foamed silicone samples in the no-PFA content state before its refilling with the required WL at the experiment’s beginning. The duration of the rejuvenation was not less than 24 h. For statistics, at least three samples of each type of the composite composition were prepared and tested.

### 2.3. Laboratory Equipment

At all stages of the work, a precise analytical balance Shimadzu ATX224 (Kyoto, Japan) and a heating cabinet ShS-80-01 SPU (SKTB-SPU, Smolensk, Russia) for the solvent evaporation were used. Also, a wide-field microscope MBS-9 (Etalonpribor, Moscow, Russia) was used for the pore examination.

### 2.4. Viscosity Measurement

The viscosity of the silicone mixtures was determined using a Brookfield rotary viscometer “Haake Vicotester 7 plus” (Thermo Scientific, Karlsruhe, Germany). The measurements were carried out at room temperature, using a spindle “L2” and a spindle rotation speed in the range from 3 to 0.2 rpm.

### 2.5. Thermal Expansion Measurement, the Device “PARUS”

The device “PARUS” was designed and made in house to measure the high coefficients of thermal expansion for soft materials, particularly for foamed composites containing in their pores a substance that undergoes a phase transition such as liquid–gas transition.

**Figure 2:**
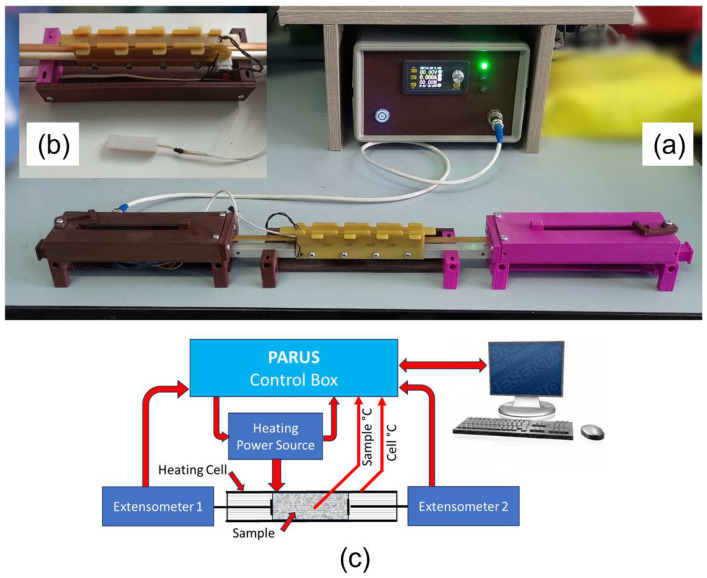
The PARUS device. (**a**) A photo of the front view of the device: control block with the heating power source and the extensometer, (**b**) the cell with a sample installed (on the top) and the sample with a temperature sensor inserted (on the bottom), (**c**) the PARUS device block diagram.

The PARUS device and its test bench with the cell are shown in Figure 2a. The PARUS device employs an external constant-power heating for a sample placed in the cell. It is controlled by the personal computer via a dedicated software developed in house. The close view on the cell is in Figure 2b. The upper lid can be removed and the sample with a thermal sensor inserted (Figure 2b) will be placed inside the cell. During the software-controlled heating, the sample is enlarged and two independent extensometers measure the length of the sample. The entire experiment is carried out in open-air conditions. The block diagram of the PARUS device is shown in Figure 2c (a detailed description of the PARUS device can be found in Supplementary Material, Figures S2 and S3).

To perform tests, the heating power for the foamed silicone samples was selected at 15–20 °C higher than the boiling point of the WL used to ensure a liquid–gas phase transition using the maximum temperature of the Ecoflex 00-50 solid silicone sample in the PARUS device cell at a different heating power (Figure S2, Supplementary Materials). The boiling temperature, cell power, and molar mass of the WL used in this work are presented in Table S1 (Supplementary Materials).

Each data point presented in the Results section is the average of at least three experiments, the calculated relative standard deviation (%RSD) of the results is always better than 17%, and in most cases, the %RSD is less than 12%. The value of the error is the sum of the uncertainness of the composite sample formation, mainly due to the random process of bubble formation in the silicone matrix and, to a lesser degree, to the PARUS device measurement results.

## 3. Results

### 3.1. Fabrication of Foamed Silicone, Viscosity During the Composite Cure

The foamed silicone composites (i.e., those having spherical isolated pores) have been demonstrated initially by A. Miriyev et al. using Ecoflex 00-50 silicone (Smooth-On, Macungie, PA, USA) and ethanol as a pore-forming additive [48]. The fabrication of foamed silicone composites could be also possible using other substances as the PFA. Because ethanol was successfully used in [48], it was decided to test in this work a homologous series of alcohols from methanol to butanol as the PFA at 20% concentration. The foamed composite was easily formed using methanol, while the other alcohols were not as successful as the PFA. When using propanol or isopropanol, the silicone cured with a significant delay and without pores, but when using butanol, almost no curing occurred.

Previously, the environmental friendliness of the foamed composite due to the use of only ethanol in the composite preparation was emphasized [48]. The use of methanol as the PFA will also not harm the environment, especially considering that the use of methanol is not mandatory during the subsequent operation of the actuator. Carrying out the rejuvenation procedure of such a composite with ethanol as the WL completely eliminates this problem.

The viscosity here reveals the solubility of the silicone matrix in the PFA liquid. Thus, the solubility of silicone increases with decreasing the alcohol polarity, which reduces the viscosity and concentration of the solution and, accordingly, the crosslinking rate. All these factors have an impact on the distribution of pores in the composite. 

**Figure 3:**
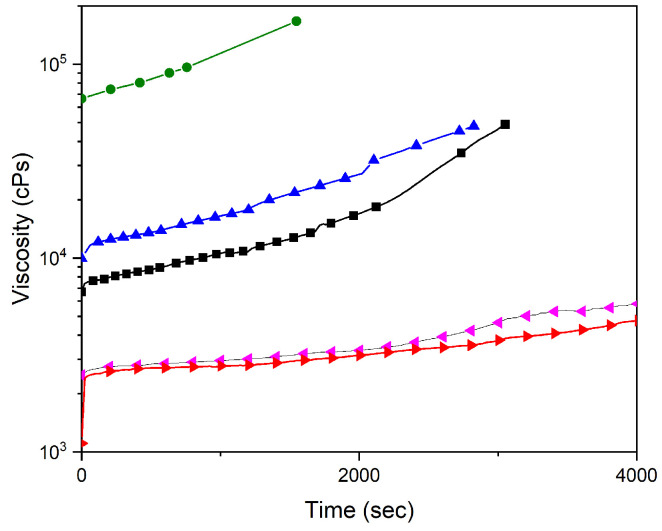
The viscosity of a mixture of silicone Ecoflex 00-50-PFA during the cure process of the composite in the presence of 20% of different alcohols used as the PFA, where ■—no PFA added, ●—methanol, ▲—ethanol, ►—isopropanol, ◄—propanol.

Changes in the viscosity during the cure of the mixture Ecoflex 00-50-PFA 20% are shown in Figure 3. In the presence of a PFA that does not significantly change the viscosity of Ecoflex 00-50 (ethanol) or increase it (methanol), the cure occurs quite quickly. In these cases, the alcohol releases from the initial mixture as drops in the volume of the silicone and remains inside the curing matrix of the composite, unable to diffuse/move outward; therefore, the pores are formed filled with the PFA liquid.

When alcohol decreases the viscosity (for propanol and isopropanol by five times at the initial stage), the curing time of the composite increases, and no pores are formed. Thus, it can be expected that not only ethanol and methanol may be suitable for the foamed composite preparation but also some other liquids or their mixtures, which are not miscible with the polymer matrix. Those liquids should ensure the preservation or increase the viscosity of the curing mixture, leading to the formation of foamed silicone, provided that there are no chemical reactions with the components of the silicone composite matrix. The mixtures 1:1 of the alcohols have been tested as the PFA (20%). If the mixture contained ethanol or methanol, pores were formed, even if half of the PFA was isopropanol or propanol. The isopropanol–propanol mixtures did not form the pores expectedly.

### 3.2. Distribution of Large Pores in the Composite

**Figure 4:**
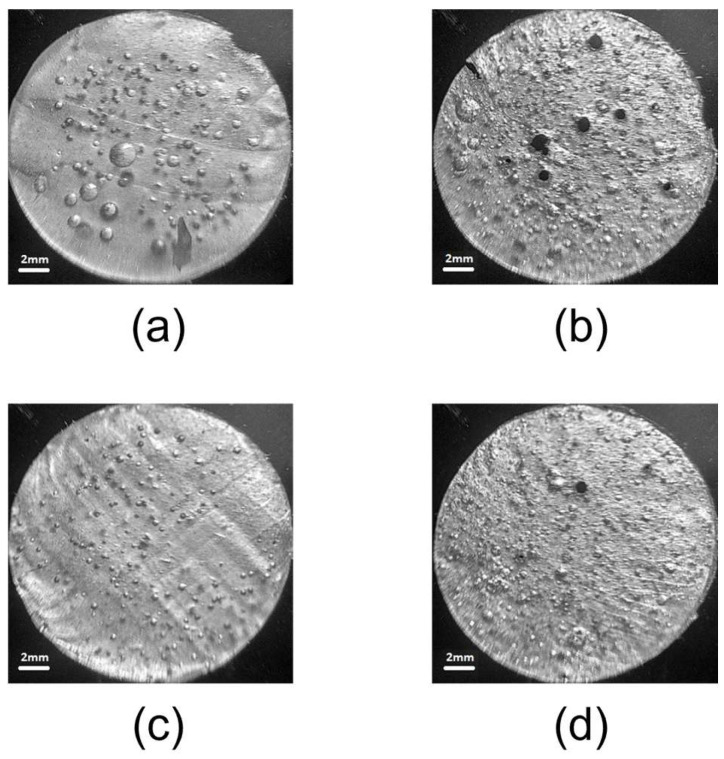
Pores in the composite prepared from different pore-forming agents: (**a**,**b**) methanol 15% and 25%, (**c**,**d**) ethanol 15% and 25%, respectively.

The distribution of relatively large pores (≈100 μm and larger) was examined using a wide-field microscope (Etalonpribor, Moscow, Russia) (see Figure 4). For this purpose, the cylindrical samples were prepared; the thickness of the slices was ~1 mm. In the composite formed using methanol as the PFA (Figure 4a,b), the pores have relatively larger sizes than those originated using ethanol as the PFA (Figure 4c,d). Moreover, they are located closer to the middle of the sample. It seems that a rapid increase in the viscosity and, accordingly, the degree of polymerization of the silicone causes methanol to be released from the matrix more intensely using the cavities of already formed pores, which leads to the formation of larger pores, and the sample surface plays also the role of the wall of the “outer” big cavity formed and the additional pores are not formed. Ethanol, having a less viscous matrix, manages to form many smaller pores distributed throughout the entire volume of the composite.

The average strain measured over all four samples is 0.36 ± 0.5. One can state that the behavior of the samples made with ethanol or methanol as the PFA is similar to each other, even though the distributions of pores both in size and position are different (Figure 4). Furthermore, below it will be shown that the amount of methanol and ethanol that can hold the foamed composite is almost equal, delivering similar strains.

### 3.3. Foamed Composite Behavior with Different Working Liquids

Albeit the pores in the silicone composite can be formed using only methanol or ethanol as the PFA, these pores can be re-filled with other WLs using the rejuvenation procedure, i.e., after drying the composite from the initial PFA and putting it into the required WL.

First of all, let us find the amount of WL that can be held by the composite made with different p-contents of ethanol as the PFA (%). According to the procedure described above, the samples prepared with different PFA contents were rejuvenated [49] using different WLs. The PFA concentrations were taken in the range of 3–35%, since at higher PFA concentrations, the joining of pores during the polymerization produces giant pores that calls into question the integrity of the composite sample.

**Figure 5:**
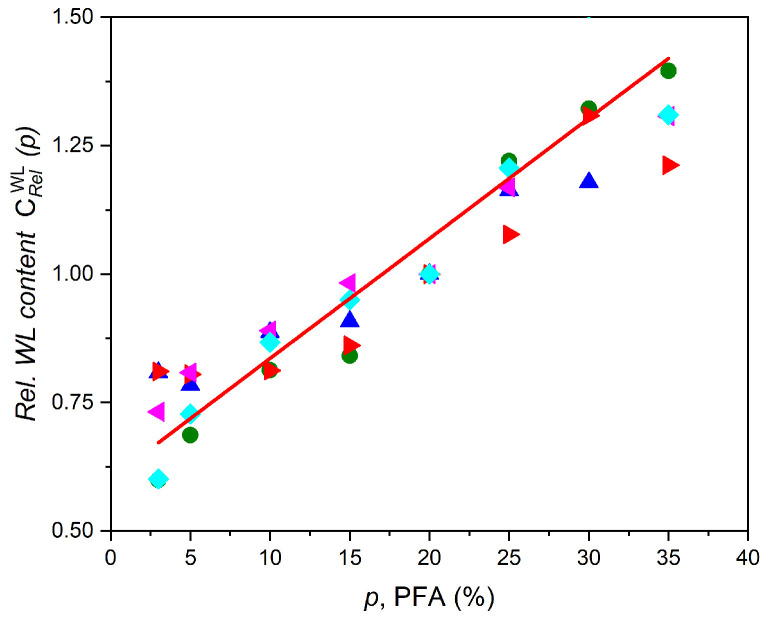
Relative specific WL content $C_{rel}^{WL}(p)$ in the composite with different concentrations of ethanol as the PFA, rejuvenated with various WLs and the linear fit of relative specific WL contents (thick red line), where ●—methanol $C_{rel}^{Methanol}(20\%)=6.8\cdot 10^{-3}$, ▲—ethanol $C_{rel}^{Ethanol}(20\%)=4.6\cdot 10^{-3}$, ►—isopropanol $C_{rel}^{Isopropanol}(20\%)=34.7\cdot 10^{-3}$, ◄—propanol $C_{rel}^{\mathrm{Pr}opanol}(20\%)=32.7\cdot 10^{-3}$, ♦—butanol $C_{rel}^{Bu\tan ol}(20\%)=39.9\cdot 10^{-3}$. The relative specific WL content set as a unit at the PFA 20%.

The WL content is $m(p)=(p)-m_{D}(p)$, where the weight of the rejuvenated sample is $m_{R}(p)$ and weight of the dried sample is $m_{D}(p)$. The specific WL content is $C^{WL}(p)=m(p)/m_{D}(p)$, accordingly, and where necessary, the upper indices will refer to the actual WL used. For the convenience of comparison, the relative specific WL content is presented as a unit of the PFA at 20%, i.e., $C_{rel}^{WL}(p)=C^{WL}(p)/C^{WL}(20\%$). This allows the reader a convenient comparison of the behavior of the samples with different WLs, while keeping in mind that the relative WL content at 20% PFA will be always equal to one. The results are shown in Figure 5, and the experimental data are given in Table S2 (see Supplementary Materials). Thus, the possibility of using different WLs (not only ethanol) for the rejuvenation procedure is shown.

The relative and consequently specific content of the WL in the composite depends almost linearly on the PFA concentration regardless of the WL used (Figure 5) due to higher pores volume formed, except for high concentrations of the PFA, where the association of pores into large and giant pores flattens this dependency. Therefore, the estimation of the $C_{rel}(p)$ behavior was made in the PFA concentrations of 3–35%. It is worth mentioning that the specific WL content is getting higher with the increasing affinity of the solvent to the silicone matrix [58].

Moreover, the density ρ(p) and the Young’s modulus E(p) of the composite within the range of 3–35% PFA were investigated. These relations are estimated as $\rho (p)=-0.02p+0.95\;g/cm^{3}$ and $E\left(p\right)=-0.01p+0.072\;MPa$ (in both cases $R^{2}>0.95$). Such a small change of ρ(p) and E(p) within the PFA range is because the presence of the pores in the matrix does not change silicone properties as soon as a relatively small volume of pores in the composite is present.

### 3.4. The Model of the Foamed Composite Behavior with Different Working Liquids

To estimate a linear deformation of the composite at conditions similar to those in the PARUS cell, the Mendeleev–Clapeyron relation [59] has been used. Let us denote the sample section as s, sample length as l, its enlargement after the liquid–gas transition as Δl, and the initial sample volume as $V_{0}=s\cdot l$. Assume, for simplicity, that the pressure P in all pores is the same, the temperature T of the sample is the boiling point temperature of the WL, and suppose that the tensile stress in the composite produced by the pressure of the WL in the gaseous phase is equal to the stress created in a mechanical tension test at any sample deformation. Keeping in mind that a relatively small volume of the composite is occupied by pores, one can say that all of the additional volume V=s·Δl of the sample is filled with a gaseous phase.

Using the assumptions described above, one can write the following:

(1) $$PV=P\cdot s\cdot \Delta l=\frac{m}{\mu }R,$$

where m—the mass of the WL in the sample, μ—the molar mass of the WL, R—the gas constant, and P,V,T—pressure, volume, and temperature, respectively.

The mechanical stress σ and strain ε=Δl/l are related in the standard linear form as σ=Eε. According to the proposed assumptions, the gas pressure should be equal to the mechanical stress detected at the same deformations. After the transformations, one obtains the following:

(2) $$\varepsilon =\frac{\Delta l}{l}=\sqrt{\frac{T}{\mu }\cdot \frac{m}{V_{0}}\cdot \frac{R}{E}},$$

where $V_{0}$—the initial sample volume (see more details in the Supplementary Materials).

The relation (2) can be divided into three separate terms: the R/E depends only on the matrix properties, i.e., in the experiments conducted, it is a constant; the second term $m/V_{0}=\rho \cdot C^{WL}(p)$ is essentially the specific WL content in the composite, which is linearly proportional to ethanol concentration (Figure 5) except for a high PFA; and, finally, the term T/μ depends only on the properties of the WL used (see Table 1). The fact that the actual temperature of the experiment is slightly higher than T does not change significantly the pressure in the pores and hence the strain behavior. Therefore, the strain is proportional to the square root of the specific solvent content $\varepsilon ∼\sqrt{C^{WL}(p)}$ for different PFA concentrations, and $\varepsilon ∼\sqrt{T/\mu }\cdot \sqrt{C^{WL}(p)}$ was used for different WLs at equal PFA contents.

The influence of the WL type on the strain of the composite at a constant PFA 20% was obtained. A set of samples (with an initial length of 40 mm) suitable for examining an actuation strain in the PARUS device were prepared with an ethanol concentration of 20% as the PFA and rejuvenated with different WLs, as described above. Table 1 shows the strain of such samples after heating to the desired temperature (ca. 15–20 °C above the boiling point of the WL used) and the calculated ratio of $K=\varepsilon /\sqrt{T/\mu \cdot C^{WL}(20\%)}$, revealing the proportionality stated by (2) for the samples with equal PFA contents.

The averaged ratio of K=2.2±0.4 (excluding the value of the isopropanol K ratio) varies in a relatively narrow span. Therefore, it could be stated that the composites under investigation behave (at least in most cases) in the manner described by Equations (1) and (2), and these relations can work as a prediction relation for a strain with the other PFAs or working liquids. The low K=0.4 ratio for isopropanol turned out to be because its ε value is close to the ethanol value, but $C^{Isopropanol}(20\%)$ is close to propanol.

### 3.5. Actuation with Different Working Liquid Content

Using Equations (1) and (2), one can find that $\varepsilon ∼\sqrt{C^{WL}(p)}$ independently on the WL used, and taking into account the results presented in Figure 5, it could be rewritten as $\varepsilon ∼\sqrt{0.02p+0.6}$ for PFA concentration. Again, because a relatively small volume of pores is present in the composite, it is difficult to observe a square root dependence, but the linearization of the relation above gives ε≈0.01p+0.79, i.e., the slope value is $S=10.0\cdot 10^{-3}$ with a determination coefficient of approximation better than $R^{2}>0.99$. Therefore, a linear dependence of strain ε from PFA concentration p is expected within the range of 3–35%.

**Figure 6:**
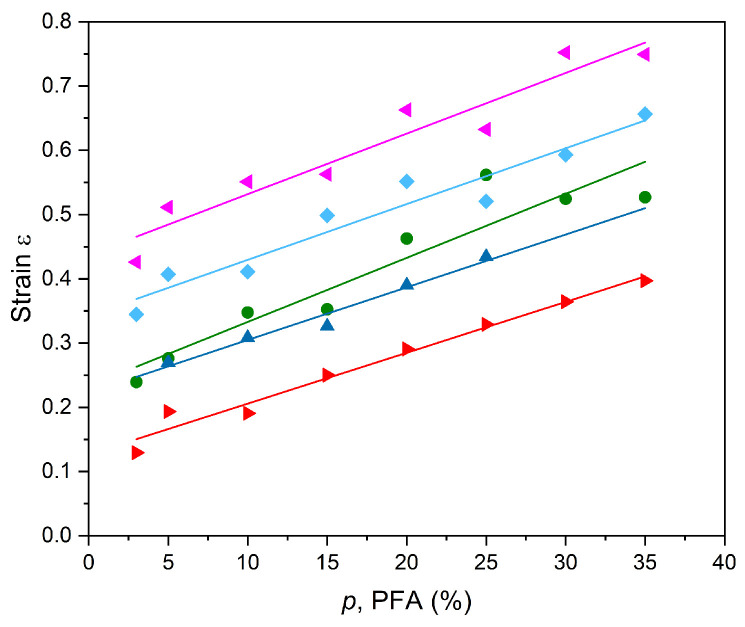
The strain of composites with different WLs in a matrix made of ethanol as PFA (%). Slopes S are as follows: ●—methanol $S=1.0\cdot 10^{-2}$, ▲—ethanol $S=8.2\cdot 10^{-3}$, ►—isopropanol $S=7.2\cdot 10^{-3}$, ◄ —propanol $S=9.4\cdot 10^{-3}$, ♦—butanol $S=8.7\cdot 10^{-3}$.

The samples for the PARUS device with different ethanol content as the PFA in the range of 3–35% were fabricated and rejuvenated according to the procedure described above. The tests were performed using the PARUS device at different heating power, where the samples had an initial length of 40 mm. The results are presented in Figure 6 and the experimental data are given in Table S3 (see Supplementary Materials).

The results shown in Figure 6 are additional experimental support to relations (1) and (2) as the strain prediction relations for the foamed composites under investigation. Indeed, the strain changes are close to linear and the calculated slopes for all the cases investigated have $R^{2}>0.9$, while the slope value is near to the above-predicted value: the calculated average slope is $S_{ave}=8.8\cdot 10^{-3}$. Isopropanol, as it was mentioned earlier for the K ratio, stands out of line having the lower $S=7.2\cdot 10^{-3}$. It is assumed that the deviation from the general values is because isopropanol has less diffusion due to branches and, therefore, less penetration during the rejuvenation process.

### 3.6. Sequential Actuation in the Open-Air Conditions

In this work, the sequential actuation of the composite is understood as the number of heating–cooling cycles in the open-air conditions until the composite is still able to actuate. Each subsequent expansion will certainly be smaller than the previous one.

To perform this type of the experiment, the PARUS device operates in a cyclic mode: heating the sample with the desired power and then cooling the cell until near-to-room temperature. Heating (and cooling) are continued until the sample length within the last 120 s is not changing to more than 1 mm, i.e., the composite reaches its maximum strain (or is close to it). Different heating-off conditions, as well as conditions different from those in the cell of the PARUS device, will give some differences in the WL losses from the composite and therefore in the cycle number. However, here such conditions will be used for all the samples to allow us to compare the possibility of the sequential actuation at different PFA % (ethanol) and for the different WLs used within this work.

**Figure 7:**
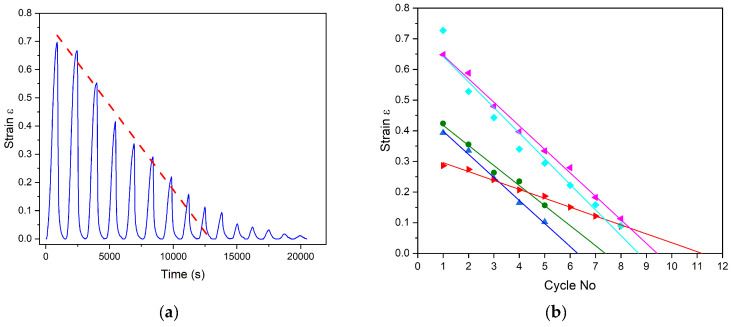
A typical behavior of the strain of the composite sample during the cyclic testing (PFA 20% ethanol): (**a**) the strain for the sample with WL propanol is shown with linear peak top fitting line (red dashed line), (**b**) the averaged strain of composites with different WL for the cyclic testing with their linear approximation showing the X intercept. ●—methanol, ▲—ethanol, ►—isopropanol, ◄—propanol, ♦—butanol.

The peculiarity of the operating conditions of the actuator in open air is the loss of the WL during the heating of the composite because of the high diffusion of the working liquid through the silicone matrix. Figure 7a shows the cycling strain of a sample with 20% PFA and WL propanol.

Indeed, Figure 7a presents a typical behavior of the composite. The amplitudes of the first cycles decrease linearly with the sequential actuation number, and then the actuation strain becomes small, close to zero value. Therefore, to find the number of open-air actuations, the initial linear part of peak tops is extrapolated to a zero-strain value (see dashed line in Figure 7a) to find the number of actuation cycles possible for a certain PFA (%)-WL combination. For the presented sample, presented in Figure 7a, the total number of cycles is 9.

The averaged behavior of the composite with different WLs and PFAs is presented in Figure 7b. The time axis intersection of the linear interpolation of sequential amplitudes of cycling testing for each WL is easily seen in Figure 7b. In Table 2, the numbers of the actuation cycles are presented for three PFA (%) concentrations, ethanol; 10%, 20%, and 30%, and the different WLs used within this work are rounded to the nearest integer.

The data in Table 2 reveal that the WL leaves the composite with similar speed almost independently upon the PFA concentration, i.e., the diffusion through the silicone matrix is the main source of the WL loss, but anyway, the amplitudes of the strain peaks depend upon the PFA (see Figure 6). The better result demonstrates isopropanol withstanding 11 sequential actuations, propanol and butanol show fewer sequential actuations at 10 and 9, respectively, and the mediocre results are for ethanol and methanol with 6 or 7 actuations. As a result, a general trend is that the composites holding WLs with a higher molecular mass, i.e., having the lower diffusion coefficients, withstand more actuation cycles. Interestingly, the slope of the curves remains the same for linear alcohols (methanol–butanol), while isopropanol shows a different slope. This tendency is likely due to the lower diffusion rate of isopropanol caused by its branching. The authors believe that this factor also influences the value of the term K (as shown in the Table 1) and the slope S (see Figure 6).

One of the challenges still to be solved in the future is increasing the number of heating–cooling cycles, and although it cannot be as large as those tested in a sealed block-force mode, this parameter could be improved in the future, for instance, by introducing some fillers into a silicone matrix or creating the other barriers, preventing the diffusion of alcohol used as a working liquid outside the composite.

## 4. Discussion

In this work, the behavior and the control of foamed silicone composite actuators employing liquid–gas phase transition by both the actuation temperature and the degree of expansion of the actuator in the open-air were investigated.

For the first time, the foamed silicone composite formation using methanol was demonstrated, while the application of the other alcohols with relatively low boiling temperatures, such as propanol, isopropanol, and butanol, did not allow the formation of a foamed structure of the composite samples due to their better compatibility with the silicone matrix.

From the point of view of environmental safety, methanol does not pose a significant increase in danger.

This work has also demonstrated novel working liquids for this type of actuators, among which are methanol, propanol, isopropanol, and butanol, undergoing a liquid–gas phase transition in the silicone actuator composite. Filling and restoring the working liquid content of the actuator was performed by applying the rejuvenation procedure using the required working liquid. It was found that the specific content of the working liquid in the actuators linearly depends on the concentration of ethanol that is used as a pore-forming agent.

As a result of the assessment of the degree of expansion of actuators during heating and the liquid–gas transition using an ideal gas law, it is theoretically predicted that the expansion of the composite is proportional to the specific content of the working liquid for composites made with pore-forming agent content in the range of 3–35%. This was confirmed experimentally using the PARUS, a made in-house device tailored for the purpose to test the materials with high degrees of thermal expansion in both single and cyclic heating actuation settings.

The cycling actuation was used to test the open-air performance of the fabricated actuators. It was found that working solvents with higher molar weights and hence lower diffusion in the silicone matrix demonstrated prolongated functionality.

## 5. Conclusions

The results reported show for the first time that it is possible to separate the functions of a liquid in the silicon-based thermomechanical actuators into a pore-forming agent (PFA) and a working liquid (WL), which can be the same or different, with alcohols C1–C4 being non-mixable with the silicone matrix. The variation in the WL and the specific content of a PFA during the composite cure makes it possible to control the main characteristics of the actuator operation temperature and degree of expansion.

The WLs used within this work allowed varying the actuator operation temperature from 65 to 117 °C, even though the silicon matrix can withstand the higher temperatures. In a single actuation open-air condition, the strain of the composite depends on the pore-forming agent content in a linear manner with the average slope $S_{ave}=8.8\cdot 10^{-3}$. In this work, the maximal strain was demonstrated by propanol and butanol as the working liquids. For the case of sequential actuation, the rule is proposed regarding how to determine the number of effective actuations by the extrapolation of the initial linear part of the actuation strain peaks to a zero strain. If both the PFA and WL of the composite are ethanol, as it was in all previous investigations, the number of sequential actuations in the open-air conditions is 6, but changing the working liquid to isopropanol extends the number of sequential actuations up to 11, that makes the actuator lifetime almost twice higher. Additionally, the strain of the actuation increases. Generally, the replacement of the working liquid from C2 to C3–C4 alcohols allows the composite to outperform commonly used ethanol-filled composites in a number of sequential operations and strains due to the fact that C3–C4 alcohols have higher molar weights and boiling points, which leads to their slower diffusion through the silicone matrix.

Thermomechanical actuators based on the composite employing a liquid–gas phase transition are promising for use in many different applications, among them are safety valves or thermoactuated switches, and the actuation of mechanisms similar to industrial robot hands or prosthetic appliance, controlled horizontal or vertical movement units, soft but powerful grips or jaws, etc. The results obtained in this work, both theoretical and experimental, pave the way to overcome the known shortcomings of their operations in the future.

## Figures and Tables

**Table 1:** Parameters of the samples with ethanol concentration of 20% as PFA, calculated as K—the ratio of strain to specific WL content.

Working Liquid	Strain ε=Δl/l	$\frac{T}{\mu }$	$C^{WL}(20\%)$	K
	at PFA 20%	°K·kg/mol	kg/m^3^·10^−3^	×10^−3^
Methanol	0.42	10.56	6.8	2.5
Ethanol	0.39	7.63	4.6	1.8
Isopropanol	0.29	5.93	34.7	0.4
Propanol	0.65	6.22	32.0	2.1
Butanol	0.73	5.28	39.9	2.5

**Table 2:** The number of actuation cycles for the samples with PFA ethanol (%) and different WLs.

WL: PFA (Ethanol)	Methanol	Ethanol	Isopropanol	Propanol	Butanol
10%	6	5	9	8	8
20%	7	6	11	9	9
30%	7	6	11	10	9

## Data Availability

Data available on request.

## Supplementary Materials

The following supporting information can be downloaded at: https://www.mdpi.com/article/10.3390/polym17010020/s1, Preparation of foamed silicone, Figure S1: Detailed description of the PARUS device; Figure S2: The PARUS cell temperature; Figure S3: Results of the PARUS device test; Table S1: Working solvents., Experimental data, Table S2: Relative specific WL content; Table S3: The strain of composites with different WL.
