# Supplementary material for: Liquid–Gas Phase Transition Actuator: Rejuvenation Procedure Extended and Open-Air Performance
Source: Polymers (Basel). 2024 Dec 25;17(1):20. doi: 10.3390/polym17010020 (PMC11722774; doi:10.3390/polym17010020)
Supplement: Supplementary file 1 [file polymers-17-00020-s001.zip › polymers-3321742-supplementary.pdf]

Supplementary Material

Liquid-gas phase transition actuator: rejuvenation procedure extended and open-air performance

Igor Bezsudnov, Alina Khmelnitskaia, Aleksandra Kalinina, Kristina Monakhova, Sergey Ponomarenko

Enikolopov Institute of Synthetic Polymeric Materials of Russian Academy of Sciences, Profsoyuznaya Str. 70, 117393 Moscow, Russia; bezsudnov_iv@ispm.ru (I.B.); alina.khmelnitskaya@ispm.ru (A.X.); kalinina@ispm.ru (A.K.); k.myagkova@ispm.ru (K.M.); ponomarenko@ispm.ru (S.P.)

Preparation of foamed silicone

The Ecoflex 00-50 components A and B are to be mixed at the 1:1 ratio (Figure S1 (a), Supplementary Material). To fabricate the foamed composite material, the silicone component A was manually stirred for 1 min with PFA (pore forming agent) in the amount of 3, 5, 10, 15, 20, 25, 30, and 35 vol.% relative to the full A+B composite volume (Figure S1 (b)), then manually mixed with the component B for 1 min (Figure S1 (c)). The mixture was cast into molds made by a 3D printer using ABS (acrylonitrile butadiene styrene) or PLA (polylactic acid) polymer (Figure S1 (d)). Two types of molds were used: 20 mm dia. mold (for porosity investigation) and
10 × 10 mm section mold with the 40 mm length for use in the PARUS device (see below) for expansion testing.

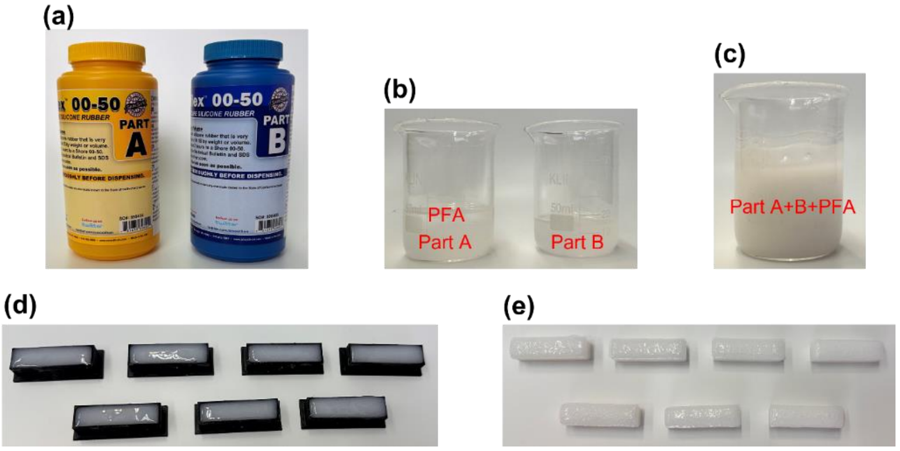

Figure. S1. Preparation of foamed silicone. (a) Two-component PDMS Ecoflex 00-50 (Smooth-On, PA, USA); (b) Component A with PFA and component B; (c) Mixture of components; (d) The casting in molds; (e) Ready-made silicone composite.

The cast material takes up at least three hours to cure at room temperature. Once the silicone had cured and the ready specimen was removed from the mold, it was processed overnight in the heater cabinet

at a temperature of 15 °C less than the boiling point of the PFA used to initially remove the preparation solvent from the pores of the composite (Figure S1 (e)).

Detailed description of the PARUS device

The PARUS device and its cell are shown in Figure 2. The device is controlled by the Arduino Nano v.3 microcontroller, the block diagram is shown in Figure 2c. The control program was created in the Arduino IDE v1.8.19 development environment. The host computer is connected to the instrument via the USB port; in the manual mode, the PARUS device is controlled via a monitor of the connected serial port using one-character commands, in the automatic mode – by the specially developed software running in the MS Windows™ environment.

The PARUS device employs an external heating for a sample placed in the cell, see Figure 2b. The cell has a square cross-section of 10×10mm. The walls are made of a glass-reinforced epoxy material FR-4 usually used for PCB manufacturing, with a thickness of 1.75 mm, a copper thickness of 35 µm, and a maximum operating temperature of 175 °C, the copper side is inside the cell. The heater is a copper meander etched on the inner surfaces of the cell walls, the width of the conductor is 0.25 mm, and the total length of the conductor is about 7 m. All the composite samples have a cross-section of 10×10 mm and a length of 25 - 50 mm.

To power the cell heater, a cascade of two power sources is used: the unregulated power supply model LRS-100 (Mean Well, Taiwan) with an output voltage of 48V, current 2.3A, and the microprocessor-controlled power supply model DSP5005 (Gaqqee, China), the output voltage range is 0-45V and the current up to 5A, the accuracy of voltage/current setting is 0.01V / 0.001A. In this work we perform heating using a constant power, the maximal power supplied to the cell heater can be up to 30W. Every 15 sec the supplied power is checked and corrected.

Two types of temperature measurements were realized. The temperature of the heated internal wall surface of the cell, i.e., the temperature of the sample surface, is measured by the resistance of the cell copper heater. The actual thermal coefficient is specified in the code. The temperature measurement accuracy is ±1.5 °C. The PARUS device also measures the temperature inside the sample volume, it uses a temperature sensor model NTC-100 with a measurement range of -50 ±260 °C, resistance at 25 °C 100kΩ ± 1%, and the temperature measurement accuracy is better than 0.5 °C. This sensor was placed in the center of the composite sample through a puncture made with a thin awl to the middle of the sample (Figure 2b), where the composite sample is shown with a temperature sensor inserted.

The sample elongation sensors (extensometers) measuring a distance of up to 90 mm were manufactured for this device based on a pre-calibrated sliding variable resistor with a nominal value of 10 kΩ, length of 100 mm, measurement accuracy of 0.2 mm for whole measurement range. The instrument uses two identical extensometers. When measuring the resistance of extensometers, a highly stable ADC ADS1115 with an

internal voltage reference is used. Three LED indicators and a buzzer indicate the status of the PARUS device.

Figure S2 shows the maximum temperature of the Ecoflex 00-50 solid silicone sample in the PARUS device cell at different heating power. The data make it possible to choose heating power for the foamed silicone samples when using various working liquids (WL) to ensure a liquid-gas phase transition. The power was chosen in such a way that the temperature turned out to be 15÷20 °C higher than the boiling point of the WL used. We have used such WL, which do not swell the silicon matrix having boiling point less than 120 °C, albeit the higher temperatures can be employed as well.

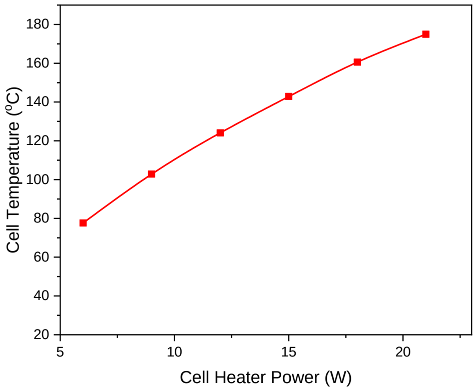

Figure S2. The PARUS cell temperature at the different cell heating power applied.

Table S1 presents working liquids used in this work together with their boiling temperature, cell power, and some other parameters.

Table S1. Working liquids: boiling temperature, molecular weight, cell heater power.

Working Liquid	Boling Temp. (BT)	Molecular weight (MM)	Heating power (W)
Methanol	65	32.0	6.0
Ethanol	78	46.1	9.0
Isopropanol	82	60.1	9.0
Propanol	97	60.1	12.0
Butanol	117	74.1	15.0

To illustrate the PARUS device operation, the results of a single measurement are shown in Figure S3, where the sample used had the ethanol as PFA with 20% concentration, WL is ethanol, the heating power is 9W, the sample length is 40 mm, the total experiment time was 800 sec. Figure S3a presents both cell temperatures during the heating, i.e., the temperature of the sample surface and the temperature inside the sample.

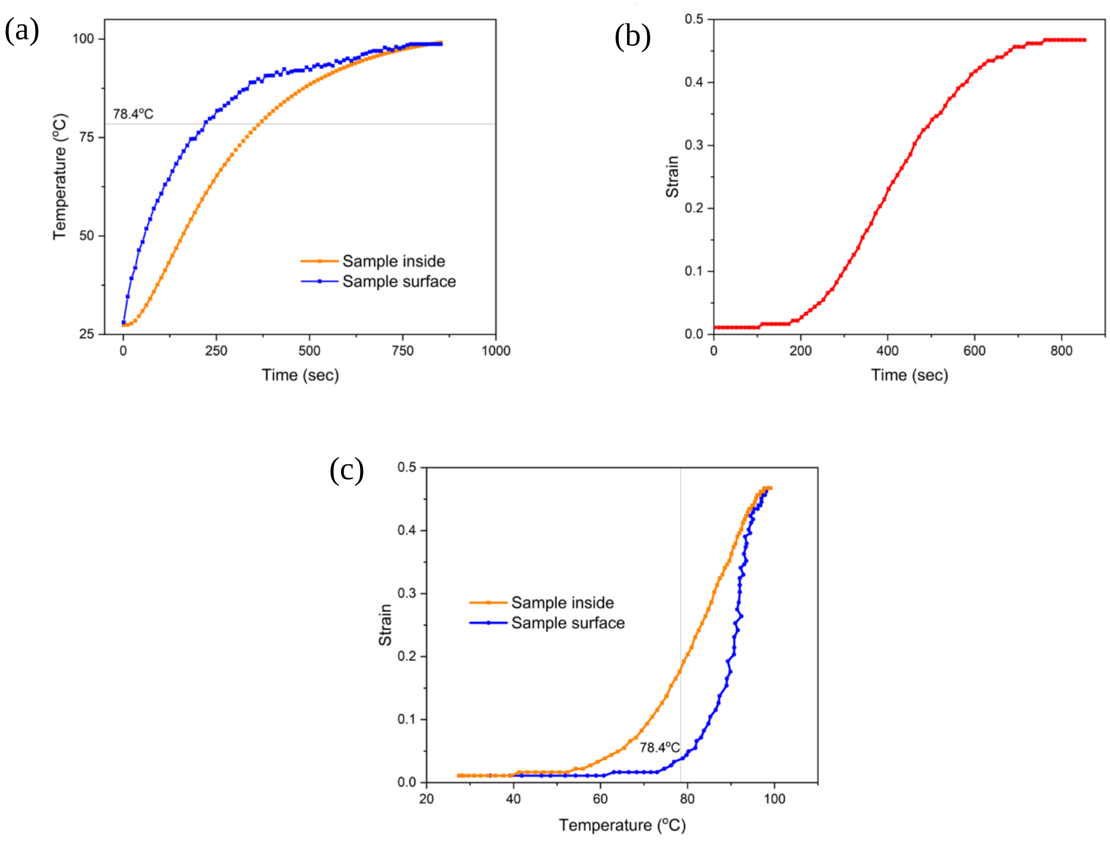

Figure. S3. Results of the PARUS device test for the composite with 20% ethanol as PFA, WL - ethanol, the heating power 9W: (a) Temperatures of the sample surface and inside the sample *vs* time; (b) Strain *vs* time, (c) Strain as a function of the inside and surface temperatures (indicated in the figure).

Since the thermal conductivity of the composite is relatively low, the temperature inside the sample turns out to be noticeably lower than on the outer heated surface until thermal equilibrium is reached in the sample-cell-environment system. In the experiment described, an equilibrium temperature was about 100 °C, and the temperature difference between the surface and the volume of the sample reached 17 °C (Figure S3a) at the moment of the extension initiation.

In Figure S3b the sample expansion at the time of the experiment is presented, where the sample stops expanding after reaching the thermal equilibrium.

Thus, during the heating of the sample in the PARUS device, its expansion (originated by a liquid-gas transition) begins in the near-surface layers, as soon as the temperature becomes higher than the boiling point of ethanol (78 °C), starting at about 180 s. From approximately the 3rd or the 4th minute of the experiment, a noise of the ethanol boiling sound can be heard. At this time, the temperature in the center of the sample (Figure S3a) is only about 60 °C, i.e., ethanol is still in the liquid phase. At about 350 s the temperature in the entire volume of the sample reaches the boiling point of ethanol, and all ethanol in the composite turns into a gas phase, which will expand further inside the composite by the laws of ideal gas restricted by the deformable silicone matrix. The expansion (300-600s)

is close to linear, see Figure S3b, and near the thermal equilibrium it ceases.

In the case of internal heating, the same uneven processes of liquid-gas transition occur, however, in this case, until thermal equilibrium is reached, the internal layers of the composite around the heater are overheated, i.e., liquid-gas transition starts there, and the outer surface has a lower temperature until the thermal equilibrium is reached.

Derivation of the strain equation

Designations:

P – gas pressure,
V, V_0 – pores volume, the initial sample volume,
T – absolute temperature,
$s, l, \Delta l$ – the sample section, length and length extension,
ε, σ – mechanical strain and stress,
R – universal gas constant,
m – mass of the WL,
μ – molar mass of the WL.

Assumptions:

1. the pressure P in all pores is the same,
2. the temperature T of the sample is the boiling point temperature of WL, all WL turn into gaseous phase,
3. the gas volume V is equal to the sample volume extension,
4. the tensile stress in the composite produced by the pressure of WL in the gaseous phase is equal to the stress created in a mechanical tension test at any sample deformations.

The Mendeleev-Clapeyron equation: $PV = \frac{m}{\mu} RT$
Mechanical stress in the sample: $\sigma = E\varepsilon$

Acc. to assumption 4 one can write $E\varepsilon \cdot V = \frac{m}{\mu} RT$
and considering that $V = s \cdot \Delta l$, $\varepsilon = \Delta l / l$, $V_0 = s \cdot l$

the relation can be rewritten using additional unit term in the form

$$E\varepsilon \cdot s \cdot \Delta l \cdot \frac{l}{l} \cdot = \frac{m}{\mu} RT$$

Extracting the strain square $\varepsilon^2 = \frac{1}{s \cdot l} \frac{1}{E} \frac{m}{\mu} RT$

and grouping the other terms: $\varepsilon^2 = \frac{m}{V_0} \frac{R}{E} \frac{T}{\mu}$

The final relation form follows:

$$\varepsilon = \frac{\Delta l}{l} = \sqrt{\frac{T}{\mu} \cdot \frac{m}{V_0} \cdot \frac{R}{E}}$$

Experimental data

Table S2. Relative specific WL content $C_{rel}^{WL}(p)$ in the composite with different concentrations of ethanol as PFA, rejuvenated with various WL and the linear fit of relative specific WL contents.

PFA %	CH_3OH	C_2H_5OH	i-C_3H_7OH	1-C_3H_7OH	1-$C_4H_{10}OH$
3	0.60	0.81	0.81	0.73	0.60
5	0.69	0.78	0.81	0.81	0.73
10	0.81	0.89	0.81	0.89	0.87
15	0.84	0.91	0.86	0.98	0.95
20	1.00	1.00	1.00	1.00	1.00
25	1.22	1.16	1.08	1.17	1.21
30	1.32	1.18	1.31	1.55	1.52
35	1.40	1.58	1.21	1.31	1.31

Averaged slope using full data set is 0.022 ± 0.0013 with $R^2 = 0.87$

Table S3. The strain of composites with different WL in a matrix made of ethanol as PFA (%).

PFA %	CH_3OH	C_2H_5OH	i-C_3H_7OH	1-C_3H_7OH	1-$C_4H_{10}OH$
3	0.24	0.25	0.13	0.43	0.34
5	0.28	0.27	0.19	0.51	0.41
10	0.35	0.31	0.19	0.55	0.41
15	0.35	0.33	0.25	0.56	0.5
20	0.46	0.39	0.29	0.66	0.55
25	0.56	0.43	0.33	0.63	0.52
30	0.52	0.47	0.36	0.75	0.59
35	0.24	0.51	0.13	0.43	0.34
Slope	$10 \cdot 10^{-3}$	$8.2 \cdot 10^{-3}$	$7.2 \cdot 10^{-3}$	$9.4 \cdot 10^{-3}$	$8.7 \cdot 10^{-3}$

All calculated slope values have R2 > 0.87. Averaged slope using full data set is $S_{ave} = 8.8 \cdot 10^{-3}$.
